# Bergamottin isolated from *Citrus bergamia* exerts *in vitro* and *in vivo* antitumor activity in lung adenocarcinoma through the induction of apoptosis, cell cycle arrest, mitochondrial membrane potential loss and inhibition of cell migration and invasion

**DOI:** 10.3892/or.2021.8058

**Published:** 2021-04-19

**Authors:** Hui-Juan Wu, Hong-Bo Wu, Yan-Qiu Zhao, Li-Juan Chen, Hong-Zhi Zou

Oncol Rep 36: 324-332, 2016; DOI: 10.3892/or.2016.4833

Following the publication of the above paper, a concerned reader drew to the Editor's attention that a number of figures (specifically, Figs. 6, 8, 9, 10 and 12) contained apparent anomalies, including repeated patternings of data within the same figure panels.

After having conducted an independent investigation in the Editorial Office, the Editor of *Oncology Reports* has determined that this paper should be retracted from the Journal on account of a lack of confidence concerning the originality and the authenticity of the data. The authors were asked for an explanation to account for these concerns, but the Editorial Office never received any reply. The Editor regrets any inconvenience that has been caused to the readership of the Journal.

